# Changes in cardiometabolic risk factors in transmasculine individuals over 10 years of Gender-Affirming Hormone Therapy

**DOI:** 10.1007/s40618-026-02906-3

**Published:** 2026-05-22

**Authors:** Martino Azzi, Alessandra Lami, Anita Dones, Arrigo Francesco Giuseppe Cicero, Arianna Siconolfi, Renato Seracchioli, Maria Cristina Meriggiola

**Affiliations:** 1 Division of Gynaecology and Pathophysiology of Human Reproduction, IRCCS Azienda Ospedaliero-Universitaria di Bologna, Via Massarenti 13, 40138 Bologna, Italy; 2 Department of Medical and Surgical Sciences (DIMEC), Alma Mater Studiorum – University of Bologna, Bologna, Italy; 3 Cardiovascular Medicine Unit, IRCCS Azienda Ospedaliero-Universitaria di Bologna, Bologna, Italy

**Keywords:** Transgender, Gender-affirming hormone therapy, Cardiovascular risk, Testosterone.

## Abstract

**Purpose:**

to describe 10-year changes in health measures and cardiovascular risk among transmasculine adults on testosterone-based Gender-Affirming Hormone Therapy.

**Methods:**

this is a single-centre, longitudinal cohort study of transmasculine adults who began Gender-Affirming Hormone Therapy and were followed-up for a total of 10 years according to standard clinical practice, which includes yearly visits with complete physical examinations, blood tests, and assessment of body composition. For participants aged 35 years or older, we estimated the 10-year cardiovascular risk, and then compared it with age-matched people from the general population.

**Results:**

57 participants completed 10 years of follow-up. Nearly half smoked and few were physically active. Haemoglobin and haematocrit rose early and then stabilised within usual ranges; very high values were uncommon. Fasting glucose, insulin, and blood pressure did not change meaningfully. The lipid profile worsened: LDL, total cholesterol and triglycerides increased, and HDL decreased throughout the study period. Weight, body mass index, waist circumference, and waist-to-hip ratio increased. Body composition showed higher lean mass and trunk fat, with a shift toward android fat distribution. No major cardiovascular events were recorded. One participant developed atrial fibrillation. Metabolic syndrome by female criteria became more frequent. cardiovascular risk was higher than cisgender women but similar to cisgender men at ages 35–44; at ages 45–54 it was lower than cisgender men.

**Conclusions:**

Over 10 years, testosterone-based Gender-Affirming Hormone Therapy is linked to less favorable lipid profiles and body composition patterns, supporting constant monitoring, risk-factor treatment, and lifestyle support.

**Supplementary Information:**

The online version contains supplementary material available at 10.1007/s40618-026-02906-3.

## Introduction

Gender-Affirming Hormone Therapy (GAHT) is a pivotal component of gender-affirming care and consists of different regimens tailored to individuals’ characteristics and desires. In transmasculine individuals, the cornerstone is the administration of testosterone (T): besides inducing changes in secondary sex characteristics, T produces other significant metabolic effects, such as increasing lean mass [1], polycythaemia [2, 3], dyslipidaemias [4, 5], and an increase in blood pressure [6], all of which are known to influence cardiovascular (CV) health. In addition, transgender and gender-nonconforming (TGNC) individuals also appear more likely to be overweight [7], and to exhibit lifestyle behaviours detrimental to CV health, such as smoking and alcohol consumption [8], and physical inactivity [9]. It thus no surprise that TGNC individuals have consistently been suggested to be at higher risk of CV disease [10, 11], metabolic syndrome [12], and all-cause mortality [13] compared with cisgender peers and the general population. Transmasculine individuals in particular have been shown to be at increased risk of myocardial infarction [14], which has also been quantified as 2-fold to 4-fold higher than cisgender men [15]. However, evidence on the long-term metabolic, CV, and anthropometric effects of prolonged T administration remains limited, as most available data derive from short- or medium-term follow-up studies. This study aims to address this gap through a single-centre, longitudinal cohort study with a 10-year follow-up.

## Materials and methods

### Study design and participants

This a single-centre, longitudinal, cohort study conducted from January 2000 until March 2025 within the Gender Clinic of the Division of Gynaecology and Pathophysiology of Human Reproduction, belonging to the *IRCCS Azienda Ospedaliero-Universitaria in Bologna*, Italy. Participants included transmasculine individuals who were referred to our Gender Clinic for gender-affirming care and then followed up according to standard clinical practice [16, 17] for at least ten years. All data were collected during follow-up, and analysed retrospectively.

Inclusion criteria were as follows: (1) having previously received a diagnosis of gender dysphoria according to DSM-IV (until 2013) or DSM-5 criteria by a licensed mental health professional, pursuant to current Italian regulations; (2) being at least 18 years of age; (3) being naïve to GAHT at baseline. All patients received adequate counselling on the nature of this study and freely gave their informed consent. The study was approved by the local Ethics Committee (*Comitato Etico Area Vasta Emilia Centro*, *CE-AVEC* and registered on ClinicalTrials.gov (ID NCT07187947).

### Measurements

All participants received a complete physical examination at baseline and then at each follow-up visit, including the measurement of blood pressure (BP), weight, height, hip and waist circumferences by a licensed physician or nurse. Anthropometric parameters were recorded according to standardised procedures based on the WHO STEPwise approach noncommunicable disease risk factor surveillance (STEPS) protocol [18]. Body Mass Index (BMI) and waist-to-hip ratio (WHR) were subsequently calculated.

Patients also underwent regular blood testing, and the following parameters were collected: haemoglobin (Hb), haematocrit (HCT), fibrinogen, International Normalized Ratio (INR), activated partial thromboplastin time (aPTT) ratio, fasting glucose, fasting insulin, total cholesterol, high-density lipoprotein (HDL) cholesterol, triglycerides (TG), luteinising hormone (LH), follicle-stimulating hormone (FSH), oestradiol (E2), total T, and sex-hormone binding globulin (SHBG). Steroid hormones were measured as previously described [19].

Low-density lipoprotein (LDL) cholesterol and free T were calculated by means of Friedewald’s and Vermeulen’s [20] formulas, respectively. Insulin sensitivity was assessed by means of the Homeostasis Model Assessment for Insulin Resistance (HOMA-IR). Impaired fasting glucose (IFG) was defined as fasting glucose levels between 100 and 125 mg/dL according to current recommendations [21], and so were dyslipidaemias: supraphysiological cholesterol if > 200 mg/dL, supraphysiological LDL if > 116 mg/dL, supraphysiological TG if > 150 mg/dL, and low HDL if < 40 mg/dL (male cutoff) or < 50 mg/dL (female cutoff) [22].

Body composition was assessed at baseline, five and ten years of GAHT by means of dual-energy X-ray absorptiometry (DEXA) using a GE Healthcare Lunar iDXA (version 16.10.151, Software Encore Version 16 - ME + 200351, GE-Lunar Inc., Madison, WI, USA). The following parameters were collected: total body mass, total body fat mass, total body lean mass, total truncal mass, truncal fat mass, total android (the portion of the abdomen included between the line joining the two superior iliac crests and extended cranially up to the 20% of the distance between this line and the chin) mass, android fat mass, total gynoid (the portion of legs from the femoral great trochanter, directed caudally up to a distance double of the android region) mass, gynoid fat mass, and the android/gynoid (A/G) ratio.

### Cardiovascular risk

CV risk factors, such as smoking status (active – at least 1 cigarette per day – smokers versus nonsmokers), dietary habits (self-reported omnivorous, vegetarian, vegan or other diet), physical activity (active if > 150 min/week; [23]), personal history of CV disorders, familiar history of CV disorders up to the first degree, or other predisposing conditions (i.e. thrombophilia, elevated serum homocysteine or the use of antipsychotics) were regularly assessed during follow-up visits. Metabolic syndrome was diagnosed according to the IDF criteria [24]. At the end of the observation period, for an eligible subcohort (aged 35 or onwards), the 10-year CV risk was calculated using the *Progetto Cuore* individual 10-year risk calculator [25], computing scores for both sexes. Then, CV risk scores and surrogate markers were compared with the age-matched general Italian population by accessing data from the same nationwide database [26–28]. Surrogate markers were compared with norms of both sexes, whereas CV risk scores were compared only with same-sex reference values.

### Statistical analysis

Continuous variables were reported as means ± standard deviations if normally distributed or medians [interquartile ranges] if not normally distributed, and as counts and frequencies when categorical. Longitudinal trends in continuous outcomes (excluding body composition, assessed only at baseline, 5 years, and 10 years) were evaluated using generalized additive mixed models (GAMMs) with subject-specific random intercepts and penalized B splines for time, treated as a smooth term with a maximum of six knots to avoid overfitting. Linearity was assumed when the estimated degrees of freedom (EDF) were ≤ 1 [29]. The models for the lipid profile and glucose metabolism were adjusted for age, smoking status and BMI; the model for BP was adjusted for age and smoking status; the models for SHBG and E2 were adjusted for age and BMI; and those for BMI, hip circumference, waist circumference, WHR, LH, and FSH were adjusted for age. For all smooth terms, the relative EDF, F and p value were reported.

To compare follow-up measurements with baseline values, linear mixed-effects models were fitted with time treated as a categorical variable and subject-level random intercepts. Post-hoc comparisons versus baseline were conducted with Dunnett’s correction for multiple testing. Categorical outcomes were analyzed using binomial generalized linear mixed models with random intercepts to assess longitudinal changes. Log-odds coefficients, odds ratios (ORs), 95% CIs, and Bonferroni–Holm-adjusted p-values were reported. Gonadectomy was then entered as an additional categorical covariate to evaluate its independent effects. Considering both the both the relatively low number of individuals on short-acting injectable esters and previous reports [30, 31] showing no significant differences on parameters between different T formulations, these were not entered as covariates. Fixed effects were presented as estimated coefficients (β) with their corresponding 95% CIs and p-values.

For the comparison with the general population, continuous variables were compared by means of the t-test for independent samples and contingency tables with Fisher’s exact or the Chi square test for categorical variables, followed by the computation of Cohen’s d and h to evaluate the respective effect sizes, interpreted as per [32]; the relevant 95% CIs were also reported.

A p value of 0.05 was considered statistically significant.

Statistical analysis was performed using the R Statistical Software (v4.2.3; R Core Team 2023).

## Results

### Study population

Following an initial screening of patient records, 143 individuals were deemed eligible for the study. Seventy-two (50.3%) were lost to follow-up, leaving a total of 71 (49.7%) who had effectively completed 10 years of follow-up. Of these, 57 (80.3%) gave their consent to participate and were included in the present study. Twenty-one (14.7%) participants were included in previous studies [4, 31].

Demographic characteristics, lifestyle habits, educational level and CV risk factors of the sample are shown in Table 1. Data on the different regimens of T and the number of individuals who underwent gonadectomy as GAS are summarised in Supplementary Table 1 (S1). Variations in GAHT regimens were motivated solely by patients’ preferences, and not because of health issues or age.

**Table 1:** Demographic characteristics of the sample (*N* = 57) at baseline. Data are reported as mean ± standard deviations (ranges), or counts (%)

Age at baseline	
29.1 ± 6.3 (18–47)	
Smoking status	
Active smokers	27 (47.4%)
Non-smokers	30 (52.6%)
Physical activity	
Inactive (< 150 min/week)	50 (87.7%)
Active (> 150 min/week)	7 (12.3%)
Diet	
Omnivorous	53 (93.0%)
Vegetarian	3 (5.3%)
Vegan	1 (1.8%)
Education level	
Primary education	23 (40.4%)
Secondary education	16 (28.1%)
Tertiary education	18 (31.6%)
Cardiovascular risk factor(s) or predisposing condition(s)	
Familial antecedent of cardiovascular disease	10 (17.5%)
Use of antipsychotics	3 (5.5%)
Heterozygous Factor II mutation	3 (5.5%)
Heterozygous Factor V Leiden	2 (3.6%)
Hyperhomocysteinaemia at baseline	2 (3.6%)

### Haematologic parameters

Hb and HCT increased significantly at all time points versus baseline. Throughout the whole observation period, the number of individuals with supraphysiological HCT values was low, and did not vary significantly over time. An effect of time could be detected for both PLTs and fibrinogen, but only the PLT count showed a significant decrease at years 2, 6, 7, 9 and 10 with respect to the baseline. PT and aPTT did not show significant changes over time. Data are presented in Table 2, S2, S3, and Supplementary Figure S1.

**Table 2:** Haematological parameters throughout the study period. Continuous variables are reported as mean ± standard deviation, or as median [IQR]. Categorical variables are reported as counts (%)

Parameter	Baseline	Year 1	Year 2	Year 3	Year 4	Year 5	Year 6	Year 7	Year 8	Year 9	Year 10
Hb [g/dL]	13.5 [12.7–14.0]	14.9 [14.1–15.8]*	15.1 ± 1.4*	15.1 [14.5–15.9]*	15.2 ± 1.4*	15.4 [14.8–16.1]*	15.6 ± 1.3*	15.3 ± 1.3*	15.1 ± 1.1*	15.4 [14.5–16.2]*	15.4 [14.5–15.9]*
HCT [%]	40.0 ± 3.0	45.1 [43.3–46.7]*	45.3 ± 3.6*	44.6 [42.9–46.7]*	45.2 ± 3.8*	45.7 ± 3.3*	46.3 ± 3.8*	45.8 ± 3.4*	45.0 ± 3.2*	45.8 [43.7–48.0]*	45.8 [43.8–47.3]*
Elevated HCT (> 50%)	0 (0.0%)	1 (1.8%)	1 (1.8%)	2 (3.5%)	3 (5.3%)	3 (5.3%)	4 (7.0%)	5 (8.8%)	3 (5.3%)	2 (3.5%)	2 (3.5%)
PLT [G/L]	269.0 [216.0–316.8]	256.0 [228.2–282.8]	254.0 [227.5–283.0]*	245.5 [219.0–296.8]	248.0 [229.5–295.5]	244.0 [231.0–301.0]	256.6 ± 50.6*	253.2 ± 49.5*	247.0 [217.5–273.5]	255.4 ± 53.8*	259.9 ± 56.9*
PT [INR]	1.0 [1.0–1.1]	1.0 [1.0–1.1]	1.0 [1.0–1.0]	1.0 [1.0–1.1]	1.0 ± 0.1	1.0 ± 0.1	1.0 ± 0.1	1.0 [1.0–1.1]	1.0 [1.0–1.0]	1.0 [1.0–1.0]	1.0 [1.0–1.0]
aPTT [ratio]	1.1 [1.0–1.1]	1.1 [1.0–1.1]	1.0 ± 0.1	1.1 ± 0.1	1.1 ± 0.1	1.0 ± 0.1	1.0 ± 0.1	1.1 ± 0.1	1.0 ± 0.1	1.1 ± 0.1	1.0 ± 0.1
Fibrinogen [mg/dL]	310.6 ± 54.5	269.5 [236.2–326.2]	264.0 [239.0–321.0]	248.0 [229.0–304.0]	250.0 [230.0–296.0]	277.3 ± 60.8	283.8 ± 61.4	289.4 ± 53.7	296.4 ± 58.8	280.9 [261.0–303.8]	305.0 [262.0–344.0]

Significant (*p* < 0.05) contrasts versus baseline are marked with an asterisk

### Glucose metabolism

Serum fasting glucose and insulin levels did not significantly change over time, and nor did the HOMA-IR index. None of the participants ever had fasting glucose levels exceeding 126 mg/dL, and the number of individuals with IFG was overall small, and did not vary significantly over time. Data are summarised in Table 3, S2 and S3, and displayed in Figure S2.

**Table 3:** Glucose metabolism throughout the study period. Continuous variables are reported as mean ± standard deviation, or as median [IQR]. Categorical variables are reported as counts (%)

Parameter	Baseline	Year 1	Year 2	Year 3	Year 4	Year 5	Year 6	Year 7	Year 8	Year 9	Year 10
Fasting glucose [mg/dL]	83.0 [79.0–88.5]	83.5 [78.8–87.2]	82.4 ± 5.6	81.6 ± 8.5	84.6 ± 10.9	84.7 ± 7.0	84.8 ± 9.4	84.6 ± 9.8	83.8 ± 10.4	85.0 [79.0–91.0]	85.8 ± 11.2
Impaired fasting glucose (100–125 mg/dL)	3 (5.3%)	2 (3.5%)	0 (0.0%)	0 (0.0%)	4 (7.0%)	1 (1.8%)	2 (3.5%)	2 (3.5%)	2 (3.5%)	3 (5.3%)	5 (8.8%)
Fasting insulin [µU/mL]	6.9 [5.0–9.2]	6.1 [4.0–8.9]	6.0 [4.8–8.2]	6.0 [3.9–8.1]	5.6 [4.0–8.2]	6.0 [4.0–8.2]	6.0 [4.7–10.6]	5.5 [4.2–8.7]	5.7 [4.1–8.9]	6.9 [4.2–10.0]	6.2 [4.4–9.9]
HOMA-IR	1.3 [1.1–2.1]	1.4 ± 0.7	1.2 [0.9–1.7]	1.2 [0.7–1.6]	1.2 [0.8–1.8]	1.2 [0.8–1.7]	1.4 [1.0–2.3]	1.2 [0.8–2.1]	1.1 [0.7–1.9]	1.5 [0.9–2.1]	1.4 [0.9–2.0]

Significant (*p* < 0.05) contrasts versus baseline are marked with an asterisk

### Lipid profile

**Fig. 1:**
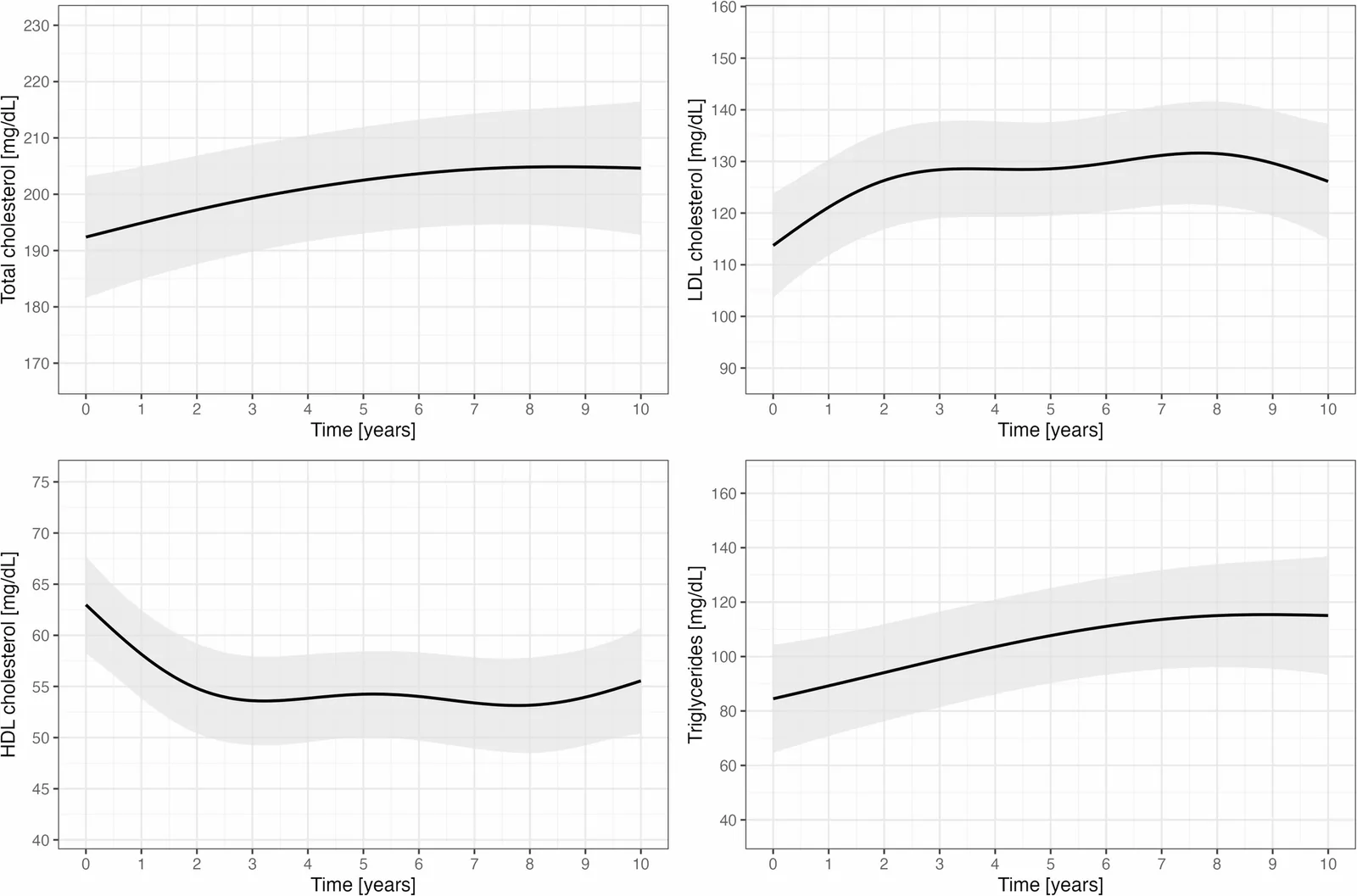
Longitudinal trend of the lipid profile. Lines represent values predicted by the generalised additive mixed models, and ribbons illustrate a 95% confidence interval

Total and LDL cholesterol, as well as TG, did show a significant increase over time, which was significant starting from the first year of GAHT versus baseline for LDL cholesterol, and from the fourth year for total cholesterol and TG. HDL cholesterol did show a significant decrease versus baseline starting from the first year of GAHT. The number of individuals with supraphysiological total cholesterol, LDL cholesterol, and TG levels also showed a significant increase throughout the observation period. Conversely, the number of individuals with low HDL did not significantly vary over time for both male and female cutoffs. During the course of GAHT, lovastatin-based dietary supplements were prescribed to 11 (21.15%) individuals, and statins were prescribed only to 2 (3.6%) individuals, not earlier than the 7th year of GAHT. Data are represented in Fig. 1, and Tables S2–S4.

### Anthropometric parameters and body composition

**Fig. 2:**
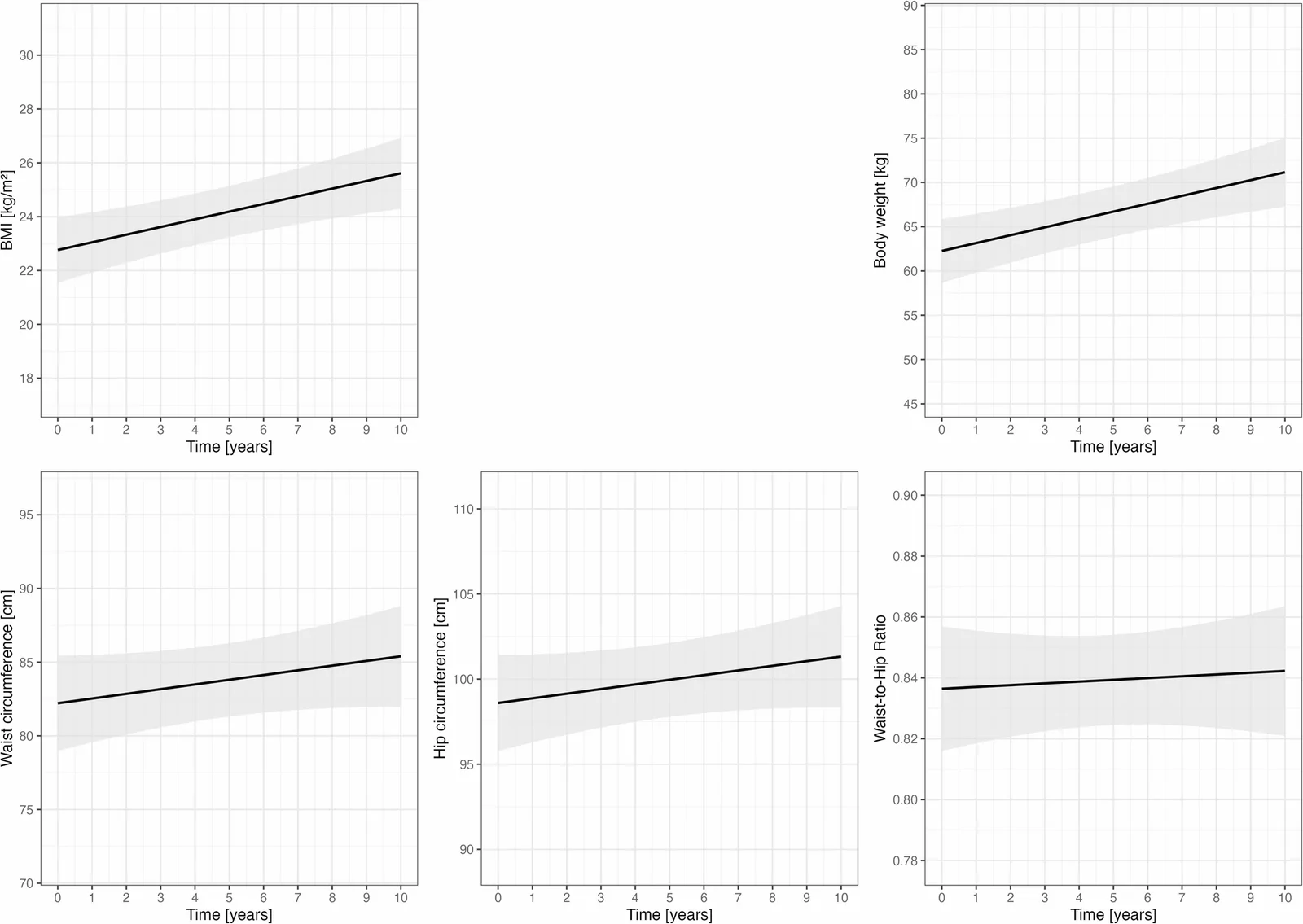
Longitudinal trend of anthropometric parameters. Lines represent values predicted by the generalised additive mixed models, and ribbons illustrate a 95% confidence interval

**Fig. 3:**
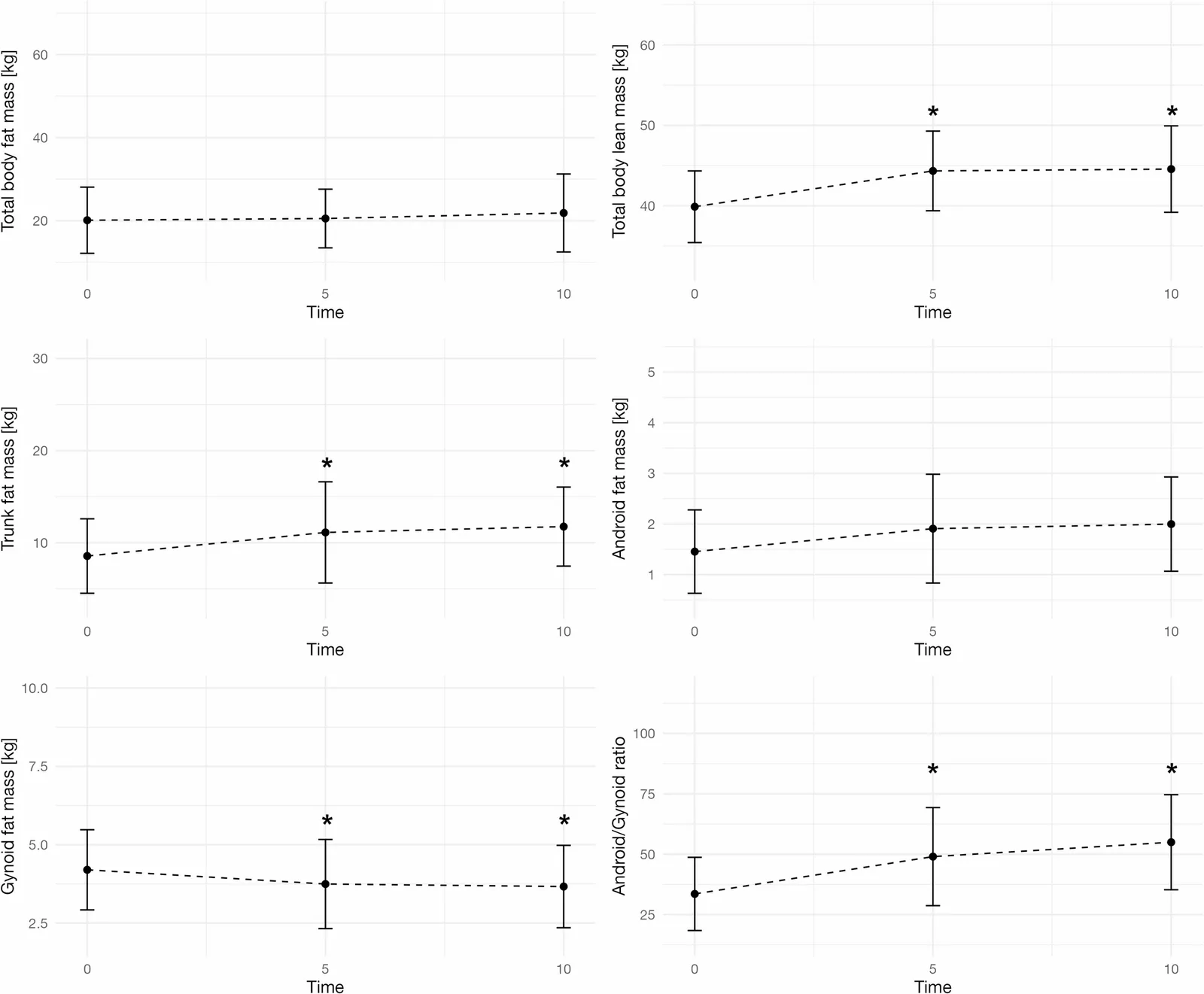
Variation of body composition over time. Dots represent means at a given time point, and bars represent standard deviations. Significant (*p* < 0.05) contrasts versus baseline are marked with an asterisk

Body weight showed a significant increase at all time points versus baseline, which however was paralleled by a significant increase in BMI only starting from year 6. Waist circumference significantly increased starting from year 6, and was paralleled by the WHR starting from the 7th year. Hip circumference, on the contrary, remained comparable to the baseline throughout the whole observation period. The number of individuals with obesity did show a significant increase throughout the observation period: however, the majority had grade I obesity, and only only one (1.8%) displayed grade III obesity at a single time point. Regarding body composition, total body lean mass, trunk fat mass and the android/gynoid (A/G) ratio did show a significant increase at all time points versus baseline. Gynoid fat mass, on the contrary, was significantly lower at 5 and 10 years versus baseline, whereas android fat mass and total body fat mass did not vary significantly throughout the observation period. Data are reported in Figs. 2 and 3, and Tables S2, S3, S5–S7.

### Hormonal profile

T and calculated free T significantly rose at all time points versus baseline, whereas E2 and SHBG decreased significantly at all time points. LH and FSH became significantly higher than baseline from year 5 and 4 onward, respectively. Data are summarised in Table 4 and S2, and shown in Figure S3.

**Table 4:** Hormonal profile throughout the study period. Continuous variables are reported as mean ± standard deviation or as median [IQR]

Parameter	Baseline	Year 1	Year 2	Year 3	Year 4	Year 5	Year 6	Year 7	Year 8	Year 9	Year 10
Total T [nmol/L]	1.4 [1.0–1.7]	15.9 [13.4–20.8]*	15.0 [12.9–20.8]*	12.3 [9.5–15.5]*	16.0 ± 8.7*	14.2 [10.1–19.1]*	14.2 [7.3–17.3]*	13.0 ± 6.1*	12.7 ± 6.7*	15.0 [9.7–21.3]*	11.9 [9.0–16.3]*
Calculated free T [nmol/L]	0.0 ± 0.0	0.4 ± 0.2*	0.3 [0.3–0.4]*	0.3 [0.2–0.4]*	0.3 [0.2–0.5]*	0.3 ± 0.2*	0.3 ± 0.2*	0.2 ± 0.1*	0.2 ± 0.2*	0.3 [0.2–0.4]*	0.3 ± 0.1*
Oestradiol [pmol/L]	392.8 [231.3–679.1]	194.6 [139.5–293.7]*	170.7 [138.6–239.5]*	168.9 [113.8–268.0]*	135.8 [69.2–189.1]*	112.0 [58.8–178.0]*	95.4 [69.8–121.5]*	88.1 [63.1–135.8]*	91.8 [56.9–124.8]*	97.3 [73.4–159.8]*	88.1 [57.8–129.8]*
LH [IU/L]	7.3 [5.2–10.8]	6.2 [2.4–9.9]	3.8 [2.1–7.5]	3.4 [1.2–7.8]	6.7 [1.8–40.9]	18.2 [5.5–35.4]*	22.6 [3.1–36.9]*	20.6 [5.9–47.8]*	26.2 [6.2–34.9]*	21.6 [5.2–35.5]*	22.9 [6.6–36.6]*
FSH [IU/L]	4.0 [3.1–5.8]	5.3 [3.9–7.2]	4.9 [2.8–6.8]	4.9 [2.2–7.8]	6.8* [3.9–63.6]	26.2 [5.5–78.7]*	36.4 [6.1–70.0]*	43.4 [12.7–86.2]*	47.6 ± 34.4*	43.0 [6.0–65.8]*	46.9 [17.4–65.2]*
SHBG [nmol/L]	60.9 ± 22.5	31.0 [25.0–36.8]*	31.7 ± 9.2*	34.9 ± 14.4*	36.5 ± 13.9*	34.8 ± 15.5*	32.7 ± 12.6*	34.4 [26.6–39.9]*	37.0 [27.9–48.8]*	36.3 ± 8.9*	35.0 [25.0–46.0]*

Significant (*p* < 0.05) contrasts versus baseline are marked with an asterisk

### The effects of gonadectomy

An independent effect of gonadectomy was observable for E2 (β = −137.20, 95% CI − 192.66 to − 81.74; *p* < 0.0001), FSH (β = 41.92, 95% CI 34.11–49.72; *p* < 0.0001), and LH (β = 22.85, 95% CI 18.14–27.56; *p* < 0.0001), as predictable; no other significant effects were found.

### Cardiovascular health

No major CV accident (i.e. acute coronary syndrome, deep venous thrombosis or pulmonary embolism) was recorded for any of the subjects during the observation period. Only one (1.8%) individual developed a supraventricular arrhythmia that later degenerated to atrial fibrillation (AF). BP values did not show significant variations over time, and nor did the number of individuals with elevated BP; throughout our observation period, only two individuals (3.6%) were prescribed antihypertensive medications. The number of individuals meeting the criteria for the Metabolic Syndrome increased significantly for the female but not for the male criteria. In the eligible subgroup (*N* = 42, mean age 41.9 ± 4.8), the 10-year CV risk estimate according to the *Progetto Cuore* score was 2.6% ± 1.1 for the male score, and 0.8% ± 0.5 for the female score. Data are summarized in Table 5, S2, S3 and S7, and pictured in Figure S4.

**Table 5:** Blood pressure values throughout the observation period. Continuous variables are reported as mean ± standard deviation or as median [IQR]. Categorical variables are reported as counts (%)

Parameter	Baseline	Year 1	Year 2	Year 3	Year 4	Year 5	Year 6	Year 7	Year 8	Year 9	Year 10
Systolic BP [mmHg]	115.1 ± 10.4	115.2 ± 10.1	120.0 [115.0–120.0]	117.2 ± 10.1	117.0 ± 7.2	115.9 ± 8.0	115.0 [110.0–125.0]	115.9 ± 9.4	120.0 [115.0–125.0]	115.0 [110.0–120.0]	115.0 [110.0–120.0]
Diastolic BP [mmHg]	70.0 [70.0–75.0]	70.0 [70.0–80.0]	75.0 [70.0–80.0]	76.5 ± 8.6	75.2 ± 7.4	70.0 [70.0–80.0]	75.0 [70.0–85.0]	70.0 [70.0–75.0]	70.0 [70.0–80.0]	75.0 [70.0–80.0]	75.0 [70.0–80.0]
Mean Arterial Pressure [mmHg]	86.7 [83.3–90.0]	86.1 ± 7.4	90.0 ± 9.5	90.1 ± 8.7	89.1 ± 6.8	85.0 [83.3–91.0]	87.5 ± 7.9	86.8 ± 7.2	87.5 [85.0–94.2]	88.8 ± 7.2	88.8 ± 6.3
Elevated BP (> 135/85 mmHg)	5 (8.8%)	1 (1.8%)	3 (5.3%)	4 (7.0%)	4 (7.0%)	7 (12.3%)	10 (17.5%)	4 (7.0%)	3 (5.3%)	4 (7.0%)	7 (12.3%)

Significant (*p* < 0.05) contrasts versus baseline are marked with an asterisk

### Comparison with the general population

When compared to the age-matched general population [26, 28], the subgroup (*N* = 28) aged 35–44 displayed significantly lower body weight, waist circumference, WHR, diastolic BP, and fasting glucose than cisgender men; when compared to cisgender women, the 35–44 subcohort displayed higher body weight and waist circumference, and lower HDL. The number of active smokers was significantly higher in our sample than both cisgender men (*p* = 0.02, h = 0.44) and women (*p* < 0.001, h = 0.62). CV risk scores were significantly higher than the female general population, whereas no significant differences were discernible when compared to the male general population.

In the older subcohort (*N* = 14), aged 45–54, significantly lower body weight, WHR, and diastolic BP were found when compared with cisgender men, and no significant differences were discernible with respect to cisgender women for these measures. The number of active smokers in our sample was again higher, but not significantly, than both cisgender men (*p* = 0.33, h = 0.28) and women (*p* = 0.07, h = 0.47). Here, CV risk scores were significantly lower than the male general population, and no significant differences were observed with respect to the female general population. No significant (*p* = 0.3) differences in smoking rates were discernible between the two sub-cohorts. Data are summarised in Table 6.

**Table 6:** Comparison of CV risk factors and scores versus the age-matched Italian general population. The p value refers to the t test for independent samples, and was subject to Bonferroni-Holm correction for multiple testing

Variable	Age group 35–44							Age group 45–54						
	Sample (*N* = 28)	Male general population (*N* = 532)	*p* value	Cohen’s d [95% CI]	Female general population (*N* = 504)	*p* value	Cohen’s d [95% CI]	Sample (*N* = 14)	Male general population (*N* = 589)	*p* value	Cohen’s d [95% CI]	Female general population (*N* = 569)	*p* value	Cohen’s d [95% CI]
Body weight	70.6 ± 9.8	82.1 ± 15.0	< 0.0001*	−0.78 [−1.19, −0.36]	64.9 ± 13.1	0.04*	0.44 [0.03, 0.85]	67.6 ± 15.3	82.7 ± 13.4	0.02*	−1.12 [−1.68, −0.57]	66.6 ± 13.2	0.90	0.08 [−0.47, 0.63]
BMI	25.1 ± 3.5	26.8 ± 4.4	0.06	−0.38 [−0.79, 0.03]	25.1 ± 5.1	0.97	0.01 [−0.40, 0.42]	24.9 ± 5.1	27.6 ± 4.1	0.25	−0.65 [−1.20, −0.10]	26.3 ± 5.3	0.66	−0.26 [−0.81, 0.29]
Hip circumference	103.0 ± 8.58	99.4 ± 8.9	0.08	0.41 [−0.01, 0.83]	98.8 ± 10.5	0.07	0.41 [−0.01, 0.83]	100.8 ± 9.4	100.1 ± 8.2	0.90	0.09 [−0.48, 0.66]	100.6 ± 10.4	0.94	0.02 [−0.55, 0.59]
Waist circumference	86.3 ± 7.2	93.0 ± 11.6	< 0.001*	−0.58 [−1.00, −0.16]	81.2 ± 12.0	0.02*	0.43 [0.02, 0.85]	85.9 ± 13.8	96.0 ± 10.9	0.11	−0.92 [−1.49, −0.35]	85.0 ± 12.9	0.90	0.07 [−0.50, 0.64]
WHR	0.8 ± 0.1	0.9 ± 0.1	< 0.0001*	−1.29 [−1.72, −0.87]	0.8 ± 0.1	0.23	0.25 [−0.17, 0.67]	0.9 ± 0.1	1.0 ± 0.1	0.01*	−1.37 [−1.95, −0.80]	0.8 ± 0.1	0.85	0.13 [−0.45, 0.70]
Systolic Blood pressure	119.1 ± 10.1	124.3 ± 12.6	0.05	−0.41 [−0.83, 0.00]	114.4 ± 12.5	0.08	0.38 [−0.04, 0.80]	120.9 ± 8.3	130.2 ± 15.8	0.02*	−0.59 [−1.19, 0.01]	122.3 ± 16.0	0.84	−0.09 [−0.68, 0.51]
Diastolic Blood pressure	74.8 ± 7.3	83.2 ± 9.5	< 0.0001*	−0.89 [−1.31, −0.47]	74.7 ± 9.0	0.97	0.01 [−0.41, 0.43]	76.4 ± 6.0	86.3 ± 10.0	0.01*	−1.00 [−1.60, −0.40]	79.3 ± 9.3	0.33	−0.32 [−0.91, 0.28]
Total cholesterol	204.4 ± 39.9	208.8 ± 42.5	0.62	−0.10 [−0.48, 0.28]	200.4 ± 40.1	0.74	0.10 [−0.28, 0.48]	224.0 ± 32.6	216.5 ± 42.7	0.74	0.18 [−0.37, 0.73]	219.5 ± 41.6	0.84	0.11 [−0.44, 0.66]
HDL cholesterol	53.3 ± 10.8	50.2 ± 12.0	0.18	0.26 [−0.12, 0.64]	62.6 ± 14.8	< 10^− 3^*	−0.64 [−1.02, −0.26]	73.8 ± 37.2	50.0 ± 11.6	0.14	1.88 [1.32, 2.44]	62.9 ± 14.8	0.66	0.70 [0.15, 1.25]
TG	109.3 ± 49.2	131.2 ± 91.9	0.06	−0.24 [−0.62, 0.14]	87.1 ± 45.9	0.07	0.48 [0.10, 0.86]	110.4 ± 65.8	143.1 ± 94.0	0.27	−0.35 [−0.90, 0.20]	100.5 ± 54.2	0.84	0.18 [−0.37, 0.73]
LDL cholesterol	129.7 ± 37.0	132.9 ± 36.3	0.66	−0.09 [−0.47, 0.29]	120.6 ± 34.2	0.31	0.27 [−0.12, 0.65]	128.5 ± 37.5	138.5 ± 36.6	0.66	−0.27 [−0.82, 0.28]	136.4 ± 36.1	0.74	−0.22 [−0.77, 0.33]
Fasting glucose	85.1 ± 11.7	93.5 ± 14.3	< 0.001*	−0.59 [−0.98, −0.20]	86.4 ± 11.8	0.73	−0.11 [−0.50, 0.28]	91.3 ± 11.0	99.7 ± 22.0	0.11	−0.39 [−0.96, 0.19]	91.0 ± 17.7	0.94	0.01 [−0.56, 0.59]
10-year CV risk (male score)	1.7 ± 0.8	1.7 ± 1.0	0.96	−0.01 [−0.44, 0.42]	N.A.	N.A.	N.A.	2.8 ± 1.3	4.4 ± 2.9	0.002*	−0.52 [−1.150, 0.045]	N.A.	N.A.	N.A.
10-year CV risk (female score)	0.6 ± 0.4	N.A.	N.A.	N.A.	0.4 ± 0.3	0.01*	0.73 [0.05, 0.40]	1.1 ± 0.6	N.A.	N.A.	N.A.	1.2 ± 1.0	0.47	−0.15 [−0.58, 0.3]

Significant (*p* < 0.05) values are marked with an asterisk

## Discussion

We present a single-centre, longitudinal study assessing the long-term effect of the administration of T on surrogate parameters of CV risk in transmasculine individuals, with data collected throughout an observation period of 10 years. No major CV or thromboembolic events were recorded. We observed an unfavourable evolution of the lipid profile and anthropometric parameters, as well as a series of inveterate lifestyle habits which are known to be detrimental to CV health. Other parameters, such as glucose metabolism and BP did not vary significantly throughout the study. When compared to the general population, our data appears to lie in between cisgender men and women, rather than strictly resembling either one of them.

None of the 57 participants developed major CV accidents or thromboembolic events during follow-up. This finding was expected, as the cohort consisted mainly of young individuals without prior cardiovascular disease or other conditions predisposing, alone or synergistically with T, to the development of significant morbidity. Moreover, participants were closely monitored during the early phase of GAHT, a period known to be critical for the occurrence of T-related adverse events [33]. This allowed clinicians to timely adjust treatment regimens, by modifying either the dosage of the transdermal formulation or the interval of administration of injectable esters. A reflection of this attitude can be observed in the prevalence of supraphysiological HCT values observed throughout follow-up, which appears to be lower than previous reports [34, 35]. In our cohort, after an initial steep increase, HCT stabilised within normal limits over time, preventing rheological alterations in blood associated with thromboembolic risk [36]. Contextually, the modest decrease in PLT count observed during follow-up may further amount as protective factor against thrombotic events, given their role in thrombus formation and amplification [37].

The lipid profile displayed a progressive worsening over time, with an increase in total cholesterol, LDL and triglycerides, accompanied by a decrease in HDL cholesterol. The negative relationship between the latter and exogenous T administration has been well characterized both in cisgender [38–40] and TGNC individuals [41–43], but may be particularly problematic for the latter given that they may assume exogenous T for a very extended period of time. However, recent data showed that cholesterol efflux capacity was not decreased in transmasculine individuals even with low serum HDL [44, 45]. If further validated, this finding could represent a protection factor of valuable importance for our understanding of the pathophysiological modifications induced by GAHT. The observed increase in LDL and total cholesterol, which confirms previous findings seen in medium and long-term follow-up studies [4, 41, 46, 47], is problematic especially if we consider their dose-dependent relationship with cardiovascular risk [48–50]. This trend, however, may parallel the one seen in the general population, where the lipid profile is known to worsen over time even when this relationship is moderated for other factors [51]. Data from the general Italian population also show a significant proportion of young adults with dyslipidaemia, with a tendency to worsen over time analogous to our findings [52, 53].

The unfavorable evolution of anthropometric parameters was expected and is coherent with the anabolic effect of T and its known effects on body fat redistribution [54]. Although we observed an increase in the number of individuals with obesity throughout time, only a negligible percentage of individuals ever exceeding grade I; our data also seem not to exceed those from the Italian general population [26, 55].

BP did not significantly vary along the course of GAHT, which is consistent with previous studies from our and other groups [4, 31, 56, 57], but not others [6, 58, 59]. This discrepancy may be accounted for by the relatively young age of our cohort, or the fact that measurements were collected exclusively during outpatient visits, which possibly do not represent real-life values.

Glucose metabolism parameters remained comparable to the baseline throughout the whole observation period. Arguably, this is the result of an interplay between the initial increase in lean mass, which may have predisposed individuals to an increased sensitivity to insulin, and the concomitant increase in the A/G ratio, whose role in insulin resistance is well established [50, 60, 61]. In any case, none of the subjects developed diabetes or had supraphysiological fasting glucose values, and the percentage of individuals with borderline fasting glucose was overall negligible.

Compared with the age-matched general population, our cohort appears to fall between cisgender men and women rather than strictly resembling either group: this is particularly true for the lipid profile, which did not appear to differ from cisgender individuals with the exception of HDL cholesterol between the younger subcohort and cisgender women, and for individuals above 45 years of age, where CV risk scores and surrogate parameters were mostly comparable to cisgender women norms. Hypothetically, individuals who began GAHT later in life may have benefited from a longer exposure to a predominantly estrogenic hormonal milieu, which has been repeatedly characterized as protective for CV health [62, 63], whereas the younger subcohort, exposed to virilising androgen levels from an earlier age and thus to a shorter duration of estrogen-associated protection, appeared to shift more rapidly toward a male-pattern CV risk profile. However, given the observational design of the present study, no causal inference can be drawn, and this hypothesis remains speculative, warranting further investigation. In addition, it is important to note that the *Progetto Cuore* calculator has not been validated in cohorts of transgender individuals; to our knowledge, however, no CV risk score has ever been validated within this population, calling for future research in this direction.

Overall, our findings indicate an increased prevalence of CV risk factors, adding adverse metabolic and anthropometric changes to the inveterate lifestyle habits detrimental to CV health. Nearly half of participants were active smokers, and most were physically inactive, consistent with previous reports showing a higher prevalence of such behaviors among TGNC individuals [7–9]. Additionally, the proportion of individuals meeting the female—but not male—criteria for the Metabolic Syndrome increased over time, corroborating earlier findings from a large cohort of US TGNC veterans [12]. Finally, worth noting in this context is that contrary to what has been described in premenopausal cisgender women [64, 65], gonadectomy did not appear to negatively affect cardiovascular risk surrogates, in agreement with prior observations reported by Grimstad and colleagues [66].

Regarding minor cardiovascular events, one individual developed a supraventricular arrhythmia that degenerated to AF. To date, evidence in transgender men is limited to a single TriNetX-based study, which found no increased risk of AF or thromboembolic events in transgender men receiving GAHT compared with untreated trans men [67]. In contrast, data from cisgender populations are less consistent. The TRAVERSE trial reported increased rates of AF and nonfatal arrhythmias in the T replacement group [68], although these findings were not replicated in a subsequent TriNetX-based analysis [69] and contrast with earlier studies suggesting that the normalisation of serum T may reduce AF incidence [70]. Overall, the available evidence on T-based therapy and AF is scant and derives almost exclusively from cisgender men, limiting its generalisability to TGNC individuals.

Our study is the first to provide information on longitudinal changes in metabolic and anthropometric parameters with a 10-year follow-up, which is of particular value considering that transmasculine individuals may take GAHT for a prolonged period of time. Our study may also be of particular value for physicians confronted with clients who present additional CV risk factors, such as older age, obesity, or other dysmetabolic conditions that warrant careful consideration and competent monitoring throughout the gender-affirmation pathway.

Nevertheless, a series of limitations should be acknowledged. First, our sample is limited in size and diversity, as it included only young individuals of European ethnicity referred to a single centre, even though this design helped decreasing the inhomogeneity of follow-up protocols. In addition, losses to follow-up and not consenting to participate to the study may have introduced selection bias. Secondly, we were unable to gather precise data on dietary habits, but this information is difficult to garner in a standardized and time-effective manner during outpatient consultations, the setting where we acquired all the information for this study. Third, the lack of a control group significantly hinders our ability to draw any causal nexus on the direct effects of GAHT on our subjects. It is also true, however, that building a randomized, controlled trial with an untreated group of transmasculine individuals is unthinkable, considering it would be unethical to withhold hormones to people who need them.

## Conclusions

Our study demonstrates that the administration of T over a period of 10 years in transmasculine individuals is associated with a worsening metabolic profile that in a longer time may increase their CV risk. In particular, the unfavorable evolution of the lipid profile and anthropometric parameters warrant regular, competent monitoring. Transmasculine individuals should receive appropriate counselling in relation to the long-term metabolic effects of GAHT, to the promotion of healthy lifestyle habits, and receive the appropriate medical interventions to address metabolic imbalances if need be.

## Supplementary Information

Below is the link to the electronic supplementary material.

Supplementary Material 1
