# Supplementary material for: Changes in cardiometabolic risk factors in transmasculine individuals over 10 years of Gender-Affirming Hormone Therapy
Source: J Endocrinol Invest. 2026 May 22;49(9):2361–74. doi: 10.1007/s40618-026-02906-3 (PMC13498505; doi:10.1007/s40618-026-02906-3)
Supplement: Supplementary file 1 [file 40618_2026_2906_MOESM1_ESM.docx]

SUPPLEMENTARY MATERIAL

| Parameter | Baseline | Year 1 | Year 2 | Year 3 | Year 4 | Year 5 | Year 6 | Year 7 | Year 8 | Year 9 | Year 10 |
| --- | --- | --- | --- | --- | --- | --- | --- | --- | --- | --- | --- |
| Individuals who underwent gonadectomy | 0 (0.0%) | 1 (1.8%) | 13 (22.8%) | 24 (42.1%) | 33 (57.9%) | 38 (66.7%) | 42 (73.7%) | 46 (80.7%) | 51 (89.5%) | 52 (91.2%) | 52 (91.2%) |
| Type of GAHT | | | | | | | | | | | |
|  | Baseline | Year 1 | Year 2 | Year 3 | Year 4 | Year 5 | Year 6 | Year 7 | Year 8 | Year 9 | Year 10 |
| T undecanoate (1g/12-16 weeks IM) | n.d. | 31 (54.4%) | 33 (57.9%) | 35 (61.4%) | 38 (66.7%) | 38 (66.7%) | 39 (68.4%) | 45 (79.0%) | 45 (79.0%) | 46 (80.7%) | 46 (80.7%) |
| T enanthate (250mg/21-30 days IM) |  | 11 (19.3%) | 11 (19.3%) | 12 (21.1%) | 10 (17.5%) | 10 (17.5%) | 8 (14.0%) | 2 (3.5%) | 2 (3.5%) | 1 (1.75%) | 1 (1.75%) |
| Esthers of T (250mg/21-30 days IM) |  | 2 (3.5%) | 2 (3.5%) | 1 (1.8%) | 0 (0.0%) | 0 (0.0%) | 0 (0.0%) | 0 (0.0%) | 0 (0.0%) | 0 (0.0%) | 0 (0.0%) |
| Transdermal T (50 mg/die) |  | 13 (22.8%) | 11 (19.3%) | 9 (15.8%) | 9 (15.8%) | 9 (15.8%) | 10 (17.5%) | 10 (17.5%) | 10 (17.5%) | 10 (17.5%) | 10 (17.5%) |

Supplementary Table 1 Individuals who underwent a gonadectomy and treatment regimens throughout the observation period. Data are presented as counts (%).

| Variable | EDF | F-value | *p* value |
| --- | --- | --- | --- |
| Fasting glucose | 1.00 | 0.43 | 0.514 |
| Fasting insulin | 1.59 | 0.42 | 0.656 |
| HOMA-IR | 1.44 | 0.26 | 0.779 |
| Total cholesterol | 1.89 | 4.22 | **0.047** |
| LDL cholesterol | 2.36 | 5.38 | **0.002** |
| HDL cholesterol | 2.71 | 6.52 | **<0.0001** |
| TG | 2.07 | 5.20 | **0.007** |
| T | 4.79 | 32.91 | **<0.0001** |
| Calculated free T | 4.20 | 11.11 | **<0.0001** |
| E2 | 2.67 | 24.18 | **<0.0001** |
| SHBG | 4.80 | 29.85 | **<0.0001** |
| LH | 3.82 | 21.24 | **<0.0001** |
| FSH | 3.10 | 35.95 | **<0.0001** |
| Systolic Blood Pressure | 1.00 | 0.07 | 0.788 |
| Diastolic Blood Pressure | 1.56 | 1.03 | 0.495 |
| Mean Arterial Pressure | 1.00 | 0.21 | 0.646 |
| Hb | 4.70 | 69.89 | **<0.0001** |
| HCT | 4.72 | 34.75 | **<0.0001** |
| PLT | 1.77 | 7.01 | **0.011** |
| Fibrinogen | 2.61 | 3.90 | **0.007** |
| PT | 1.00 | 1.50 | 0.221 |
| aPTT | 1.00 | 0.00 | 0.988 |
| Body weight | 1.19 | 95.11 | **<0.0001** |
| BMI | 1.00 | 9.89 | **0.002** |
| Waist circumference | 1.00 | 1.97 | 0.161 |
| Hip circumference | 1.00 | 1.94 | 0.165 |
| WHR | 1.00 | 0.13 | 0.723 |

Supplementary Table 2 Summary of the time smooth in the generalized additive mixed models. EDF = Estimated Degrees of Freedom.

| Variable | Log-odds coefficient | Odds Ratio | 95% Confidence Interval | *p* value |
| --- | --- | --- | --- | --- |
| Elevated HCT | 0.138 | 1.148 | 1.008 – 1.308 | 0.300 |
| Borderline fasting glucose (100-126 mg/dL) | 0.192 | 1.211 | 1.049 – 1.398 | 0.080 |
| Metabolic syndrome (female elaboration) | 0.243 | 1.275 | 1.141 – 1.426 | **<0.0001** |
| Metabolic syndrome (male elaboration) | 0.062 | 1.064 | 0.897 – 1.262 | 1.000 |
| Individuals with obesity (BMI > 30 kg/m^2^) | 0.316 | 1.372 | 1.142 – 1.648 | **0.009** |
| Elevated total cholesterol (>200 mg/dL) | 0.312 | 1.367 | 1.254 – 1.489 | **<0.0001** |
| Elevated LDL cholesterol (>116 mg/dL) | 0.289 | 1.335 | 1.219 – 1.461 | **<0.0001** |
| Elevated TG (>150 mg/dL) | 0.258 | 1.295 | 1.168 – 1.436 | **<0.0001** |
| Low HDL (<40 mg/dL) | 0.072 | 1.074 | 0.998 – 1.157 | 0.401 |
| Low HDL (<50 mg/dL) | 0.001 | 1.001 | 0.884 – 1.133 | 1.000 |
| Elevated blood pressure (>135/85 mmHg) | 0.014 | 1.014 | 0.914 – 1.125 | 1.000 |

Supplementary Table 3 Log-odds coefficients, odds ratios and their 95% CIs for the generalised linear mixed models for the evaluation of the longitudinal trend of categorical variables. p values were adjusted according to the Bonferroni-Holm procedure for multiple testing.

| Parameter | Baseline | Year 1 | Year 2 | Year 3 | Year 4 | Year 5 | Year 6 | Year 7 | Year 8 | Year 9 | Year 10 |
| --- | --- | --- | --- | --- | --- | --- | --- | --- | --- | --- | --- |
| Total  cholesterol  [mg/dL] | 175.2 ± 26.4 | 185.6 ± 32.5 | 181.1 ± 37.7 | 189.9 ± 36.7 | 193.5 ± 33.0* | 194.8 ± 37.2* | 216.0 [178.2–230.5]* | 208.1 ± 35.4* | 207.8 ± 34.9* | 210.3 ± 37.1* | 207.2 ± 37.7* |
| Elevated total  cholesterol (>  200 mg/dL) | 11 (19.3%) | 18 (31.6%) | 14 (24.6%) | 16 (28.1%) | 12 (21.1%) | 21 (36.8%) | 19 (33.3%) | 20 (35.1%) | 21 (36.8%) | 19 (33.3%) | 36 (63.2%) |
| HDL  cholesterol  [mg/dL] | 62.0 ± 13.7 | 54.0 [48.0–62.0]* | 51.9 ± 12.6* | 52.2 ± 10.9* | 56.0 ± 13.1* | 55.9 ± 10.1* | 51.9 ± 10.5* | 57.6 ± 12.8* | 53.5 ± 10.8* | 54.2 ± 11.6* | 55.0 [48.0–64.2]* |
| Low HDL (<  40 mg/dL) | 2 (3.5%) | 5 (8.8%) | 5 (8.8%) | 5 (8.8%) | 2 (3.5%) | 2 (3.5%) | 4 (7.0%) | 1 (1.8%) | 2 (3.5%) | 5 (8.8%) | 4 (7.0%) |
| Low HDL (<  50 mg/dL) | 9 (15.8%) | 16 (28.1%) | 15 (26.3%) | 14 (24.6%) | 10 (17.5%) | 11 (19.3%) | 12 (21.1%) | 12 (21.1%) | 12 (21.1%) | 12 (21.1%) | 20 (35.1%) |
| LDL  cholesterol  [mg/dL] | 97.4 ± 24.6 | 113.6 ± 30.7* | 113.4 ± 34.5* | 119.3 ± 33.8* | 122.5 ± 34.3* | 117.4 ± 35.6* | 129.5 ± 25.5* | 131.2 ± 35.0* | 131.0 ± 29.5* | 133.7 ± 35.8* | 126.9 ± 34.9* |
| Elevated LDL  cholesterol (>  116 mg/dL) | 14 (24.6%) | 24 (42.1%) | 17 (29.8%) | 18 (31.6%) | 14 (24.6%) | 24 (42.1%) | 20 (35.1%) | 21 (36.8%) | 22 (38.6%) | 19 (33.3%) | 35 (61.4%) |
| TG [mg/dl] | 69.0 [58.0–85.0] | 67.5 [52.8–111.2] | 71.0 [56.0–111.0] | 82.0 [64.0–103.0] | 81.0 [66.0–117.0]* | 87.5 [65.0–123.2]* | 108.0 [87.0–134.2]* | 97.0 [65.0–127.0]* | 95.0 [69.0–156.5]* | 109.0 [73.0–143.0]* | 95.5 [69.0–146.0]* |
| Elevated TG (> 150 mg/dL) | 2 (3.5%) | 4 (7.0%) | 4 (7.0%) | 6 (10.5%) | 5 (8.8%) | 6 (10.5%) | 8 (14.0%) | 5 (8.8%) | 10 (17.5%) | 6 (10.5%) | 13 (22.8%) |

Supplementary Table 4 Lipids throughout the study period Continuous variables are reported as mean ± standard deviation or as median [IQR]. Categorical variables are reported as counts (%). Significant (p < 0.05) contrasts versus baseline are marked with an asterisk.

| Parameter | Baseline | Year 1 | Year 2 | Year 3 | Year 4 | Year 5 | Year 6 | Year 7 | Year 8 | Year 9 | Year 10 |
| --- | --- | --- | --- | --- | --- | --- | --- | --- | --- | --- | --- |
| Body weight [kg] | 60.0 [54.0–70.0] | 65.1 ± 11.1* | 66.7 ± 10.5* | 67.8 ± 10.7* | 66.3 ± 11.4* | 63.0 [57.0–70.0]* | 69.6 ± 10.8* | 66.0 [62.0–73.6]* | 66.7 ± 9.4* | 69.4 ± 11.7* | 70.2 ± 12.0* |
| BMI [kg/m^2^] | 21.7 [20.3–24.2] | 22.9 [21.5–25.7] | 24.1 ± 3.6 | 24.4 ± 3.4 | 23.5 [21.4–25.7] | 23.7 ± 3.9* | 23.7 [22.2–27.8]* | 24.5 [22.2–25.8]* | 23.7 [22.4–25.1]* | 25.1 ± 4.1* | 25.4 ± 4.1* |
| Individuals with obesity (BMI > 30 kg/ m^2^) | 4 (7.0%) | 3 (5.3%) | 2 (3.5%) | 2 (3.5%) | 2 (3.5%) | 2 (3.5%) | 4 (7.0%) | 5 (8.8%) | 3 (5.3%) | 4 (7.0%) | 8 (14.0%) |
| Waist circumference [cm] | 80.6 ± 11.5 | 80.0 [75.8–86.2] | 85.0 ± 10.0 | 85.5 ± 9.8 | 79.5 [76.0–86.2] | 81.0 [76.0–88.0] | 85.9 ± 10.8* | 84.8 ± 10.7* | 85.0 [80.5–89.0]* | 84.1 ± 9.9* | 85.7 ± 9.2* |
| Hip circumference [cm] | 99.5 ± 8.4 | 99.0 ± 8.8 | 100.4 ± 8.2 | 102.0 ± 9.0 | 100.7 ± 9.6 | 100.3 ± 8.6 | 102.3 ± 8.4 | 99.5 [94.0–104.0] | 99.0 ± 8.5 | 101.5 ± 9.5 | 101.3 ± 9.1 |
| Hip-waist ratio | 0.8 ± 0.1 | 0.8 [0.8–0.9] | 0.8 ± 0.1 | 0.8 [0.8–0.9] | 0.8 ± 0.1 | 0.8 ± 0.1 | 0.8 ± 0.1 | 0.8 ± 0.1* | 0.9 [0.8–0.9]* | 0.8 ± 0.1* | 0.8 ± 0.1* |

Supplementary Table 5 Anthropometric measurements throughout the study period. Continuous variables are reported as mean ± standard deviation or as median [IQR]. Categorical variables are reported as counts (%). Significant (p < 0.05) contrasts versus baseline are marked with an asterisk.

| Parameter | Baseline | 5 years | 10 years |
| --- | --- | --- | --- |
| Total body fat mass  [kg]  [%] | 17.7 [14.8–23.4] (9.0–43.8)  31.8 ± 7.2 (16.0–48.1) | 20.5 ± 7.0 (8.5–38.4)  30.5 ± 6.5 (17.3–46.7) | 19.5 [17.0–25.1] (8.9–57.6)  30.9 ± 6.3 (17.2–42.2) |
| Total body lean mass  [kg]  [%] | 39.2 [36.7–44.2] (32.2–51.5)  64.9 ± 7.0 (49.0–79.4) | **44.6 [41.4–47.7]* (34.1–65.5)**  66.8 ± 6.1 (51.8–79.0) | **44.6 ± 5.4* (34.1–54.6)**  66.5 ± 5.9 (56.0–79.3) |
| Trunk fat mass  [kg]  [%] | 6.9 [6.0–10.7] (3.2–16.6)  29.3 ± 10.0 | **11.1 ± 5.5* (3.2–25.3)**  34.7 ± 13.4 (11.5–60.0) | **11.8 ± 4.3* (4.0–23.6)**  35.9 ± 10.9 (12.6–59.0) |
| Android fat mass  [kg] [%] | 1.5 ± 0.8 (0.4–2.9)  32.5 ± 12.2 (13.3–52.4) | 1.9 ± 1.1 (0.4–4.3)  34.7 ± 13.4 (11.5–60.0) | 2.0 ± 0.9 (0.5–4.4)  35.9 ± 10.9 (12.6–59.0) |
| Ginoid fat mass  [kg] [%] | 4.4 [3.0–4.7] (2.7–8.4)  41.4 ± 6.1 (33.1–54.0) | **3.7 ± 1.4* (1.9–6.6)**  **33.7 ± 7.5 (22.0–47.9)** | **3.4 [2.8–4.1]* (1.9–7.1)**  **32.2 ± 6.6 (21.3–47.5)** |
| Android/gynoid ratio [%] | 33.5 ± 15.2 (13.1–63.6) | **49.0 ± 20.3* (18.3–97.4)** | **55.0 ± 19.7* (25.9–86.0)** |

Supplementary Table 6 Body composition parameters at 5-year intervals. Continuous variables are reported as mean ± standard deviation or as median [IQR], and (range). Significant (p < 0.05) contrasts versus baseline are marked with * and highlighted in bold.

| Parameter  N = 57 | Baseline | Year 1 | Year 2 | Year 3 | Year 4 | Year 5 | Year 6 | Year 7 | Year 8 | Year 9 | Year 10 |
| --- | --- | --- | --- | --- | --- | --- | --- | --- | --- | --- | --- |
| Individuals with a diagnosis of Metabolic Syndrome according to the IDF-III criteria (Female elaboration) | 4 (7.0%) | 3 (5.3%) | 2 (3.5%) | 4 (7.0%) | 3 (5.3%) | 4 (7.0%) | 10 (17.5%) | 3 (5.3%) | 7 (12.3%) | 6 (10.5%) | 14 (24.6%) |
| Individuals meeting 3 out of 5 criteria | 3 (5.3%) | 3 (5.3%) | 2 (3.5%) | 3 (5.3%) | 2 (3.5%) | 4 (7.0%) | 6 (10.5%) | 2 (3.5%) | 5 (8.8%) | 5 (8.8%) | 12 (21.1%) |
| Individuals meeting 4 out of 5 criteria | 1 (1.8%) | 0 (0.0%) | 0 (0.0%) | 1 (1.8%) | 1 (1.8%) | 0 (0.0%) | 3 (5.3%) | 1 (1.8%) | 1 (1.8%) | 1 (1.8%) | 1 (1.8%) |
| Individuals meeting 5 out of 5 criteria | 0 (0.0%) | 0 (0.0%) | 0 (0.0%) | 0 (0.0%) | 0 (0.0%) | 0 (0.0%) | 1 (1.8%) | 0 (0.0%) | 1 (1.8%) | 0 (0.0%) | 1 (1.8%) |
| Individuals meeting IDF-III criteria for the Metabolic Syndrome (Male elaboration) | 2 (3.5%) | 1 (1.8%) | 1 (1.8%) | 2 (3.5%) | 1 (1.8%) | 0 (0.0%) | 5 (8.8%) | 0 (0.0%) | 2 (3.5%) | 3 (5.3%) | 2 (3.5%) |
| Individuals meeting 3 out of 5 criteria | 1 (1.8%) | 1 (1.8%) | 1 (1.8%) | 1 (1.8%) | 1 (1.8%) | 0 (0.0%) | 3 (5.3%) | 0 (0.0%) | 1 (1.8%) | 3 (5.3%) | 1 (1.8%) |
| Individuals meeting 4 out of 5 criteria | 1 (1.8%) | 0 (0.0%) | 0 (0.0%) | 1 (1.8%) | 0 (0.0%) | 0 (0.0%) | 2 (3.5%) | 0 (0.0%) | 0 (0.0%) | 0 (0.0%) | 1 (1.8%) |
| Individuals meeting 5 out of 5 criteria | 0 (0.0%) | 0 (0.0%) | 0 (0.0%) | 0 (0.0%) | 0 (0.0%) | 0 (0.0%) | 0 (0.0%) | 0 (0.0%) | 1 (1.8%) | 0 (0.0%) | 0 (0.0%) |

Supplementary Table 7 Individuals meeting the criteria for the Metabolic Syndrome throughout the observation period.

Supplementary Figure 1 Longitudinal trend of haematological parameters. Lines represent values predicted by the generalised additive mixed models, and ribbons illustrate a 95% confidence interval.

Supplementary Figure 2 Longitudinal trend of the glucidic profile. Lines represent values predicted by the generalised additive mixed models, and ribbons illustrate a 95% confidence interval.

Supplementary Figure 3 Longitudinal trend of the hormonal profile. Lines represent values predicted by the generalised additive mixed models, and ribbons illustrate a 95% confidence interval.

Supplementary Figure 4 Longitudinal trend of blood pressure. Lines represent values predicted by the generalised additive mixed models, and ribbons illustrate a 95% confidence interval.
